# Cytologic features of gastric-type endocervical adenocarcinoma: Three cases report and literature review

**DOI:** 10.1097/MD.0000000000040149

**Published:** 2024-10-25

**Authors:** Anchun Liu, Maiqing Yang, Hao Zou, Xiaolin Gong, Chang Zeng

**Affiliations:** a Department of Pathology, Yueyang Central Hospital, Yueyang City, Hunan Province, China; b Department of Pathology, Weifang People’s Hospital (First Affiliated Hospital of Shandong Second Medical University), Weifang City, Shandong Province, China; c Department of Radiotherapy, Yueyang Hospital of Traditional Chinese Medicine, Yueyang City, Hunan Province, China.

**Keywords:** cervix, cytology, gastric-type adenocarcinoma, minimal deviation adenocarcinoma, mucinous adenocarcinoma, three-dimensional spheroids

## Abstract

**Rationale:**

Cervical gastric-type adenocarcinoma shows gastric differentiation, and the tumor cell morphology appears benign and unrelated to human papillomavirus, which makes cervical gastric-type adenocarcinoma highly susceptible to misdiagnosis as normal glandular epithelium in cytologic diagnosis.

**Patient concerns:**

We present 3 cases of gastric-type adenocarcinoma, with the first being a 57-year-old female with abnormal uterine bleeding and fluid drainage. The second patient was a 63-year-old female, and the third was a 59-year-old female with irregular vaginal bleeding after menopause.

**Diagnosis:**

The 3 patients were diagnosed with cervical gastric-type adenocarcinoma by combining their history, clinical data, cytopathology, histopathology, and immunohistochemistry.

**Interventions:**

Patient 1 underwent total hysterectomy and adnexectomy, but refused radiotherapy and chemotherapy. Patient 2 received a chemotherapeutic regimen, and patient 3 was treated with traditional Chinese medicine.

**Outcomes:**

Patient 1 was followed-up for 9 months and is currently in good general condition, while patients 2 and 3 were not followed-up.

**Lessons:**

The “drunken honeycomb” cell arrangement is diagnostically important in liquid-based cytology, especially when three-dimensional spheroids are present, and may be a characteristic cytological finding of well-differentiated cervical gastric-type adenocarcinoma.

## 1. Introduction

Cervical gastric-type adenocarcinoma (GAS) encompass a well-differentiated adenocarcinoma that has been historically termed “minimal deviation adenocarcinoma” (also adenoma malignum).^[1]^ Minimal deviation adenocarcinoma accounts for 1% to 3% of adenocarcinomas of the cervical canal and is defined as a highly differentiated mucinous adenocarcinoma, with indistinguishable cytologic features from benign cervical glandular epithelial cells, although its clinical behavior is usually invasive.^[2]^ GAS is morphologically distinguished by tumor cells with distinct cell borders with pale eosinophilic cytoplasm that appear morphologically similar to pancreato-biliary adenocarcinomas.^[3]^ Liquid-based cytology (LBC) has become a common screening method for cervical cancer, and despite the typical histologic features of GAS, cytologic diagnosis is often challenging, and there is a paucity of data on the characteristics of GAS liquid-based cytology.^[4]^ Our case report analyzes the cytological features of 3 patients with GAS and reviews relevant literature.

## 2. Case presentation

### 2.1. Ethic approval

This study was approved by the Ethics Committee of Yueyang Central Hospital. The ethics committee approval number is 2024-015. Written informed consent was obtained from 3 patients for publishing this case report and accompanying images.

### 2.2. Case 1

**Figure 1.**
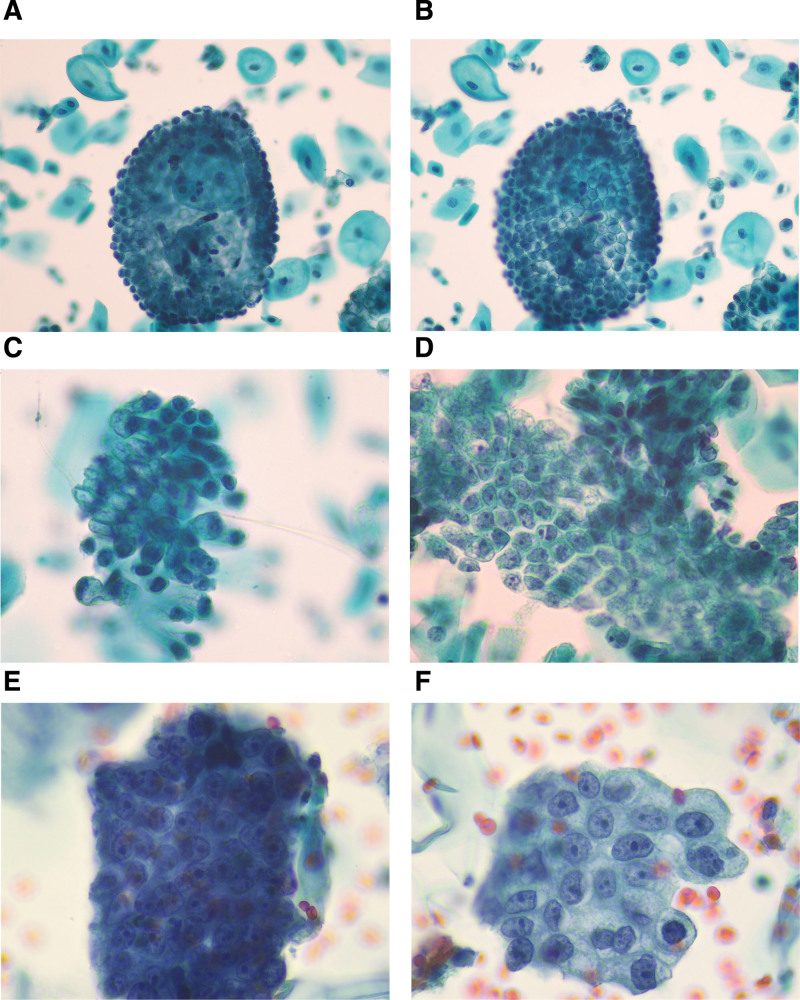
LBC samples of patient 1. (A–D) The first LBC sample of patient 1. With “drunken honeycomb” patterns of different tumor cell arrangements. (A) Tumor cells were arranged in three-dimensional stereosphere. By adjusting the focusing plane of the microscope, the nucleus pole of tumor cells could be observed (Papanicolaou, Pap staining, 200× magnification). (B). The mucin pole of tumor cells could be observed (Pap staining, 200× magnification). (C). Tumor cells were arranged in clusters (Pap staining, 400× magnification). (D). Tumor cells were arranged in a monolayer with uneven cell distribution and unequal spacing of nuclei (Pap staining, 400× magnification). (E and F) The second LBC sample of patient 1. (E) The tumor cells were arranged in clusters, and the nuclei of the tumor cells were crowded and enlarged (Pap staining, 400× magnification). (F) The tumor cells were arranged in single flat layer, with vesicular chromatin, prominent nucleoli, and thickening and irregularity of the nuclear membrane (Pap staining, 400× magnification).

A 57-year-old woman presented with abnormal uterine bleeding and vaginal discharge. Her human papillomavirus (HPV) test results were negative, and the initial diagnosis of cervical LBC was negative for intraepithelial lesions or malignancy. Microscopically, the cells were arranged in clusters, monolayers, and three-dimensional spheres. A large number of glandular cells were highly columnar, with abundant mucin in the cytoplasm, a low nuclear-to-plasma ratio, and well-defined cytoplasmic borders (Fig. 1A–D). Cervical magnetic resonance imaging (MRI) showed an enlarged cervical canal, and a mixed-signal mass shadow of about 4 cm × 3.1 cm × 3.5 cm in size was observed in the cervical canal. The lesion infiltrated the muscular layer of the uterine cervix, and no obvious breakthrough of the plasma membrane layer was seen. Therefore, the patient underwent a cervical canal biopsy, a second LBC was performed on the upper part of the cervix, and diagnosed as atypical glandular cells-favor neoplastic (AGC-FN). Microscopically, the tumor cell nuclei were enlarged and crowded, with unequal spacing of the nuclei, thickening, and irregularity of the nuclear membrane, and significant nucleoli (Fig. 1E–F). The histopathological diagnosis of the cervical canal tissue was HPV-independent gastric-type cervical adenocarcinoma. Microscopic examination revealed irregularly shaped, angulated cervical glands, and the tumor cells grew infiltratively into the cervix, with reactive hyperplasia of the surrounding fibrous interstitium. The glandular cytoplasm was rich in mucus and clear or pale eosinophilic. Immunohistochemistry revealed MUC6 (+), P16 (weak+), CK7 (+), CEA (+), CDX-2 (+), P53 (mutant type), PAX-8 (+), and ki-67 (20%, +), while CK20, SATB2, ER, HNF-1β, and Napsin A were negative. The patient underwent total hysterectomy and adnexectomy, did not receive any postoperative radiotherapy or chemotherapy, and recovered well from the operation. At the nine-month follow-up, no recurrence nor other metastatic diseases were observed.

### 2.3. Case 2

**Figure 2.**
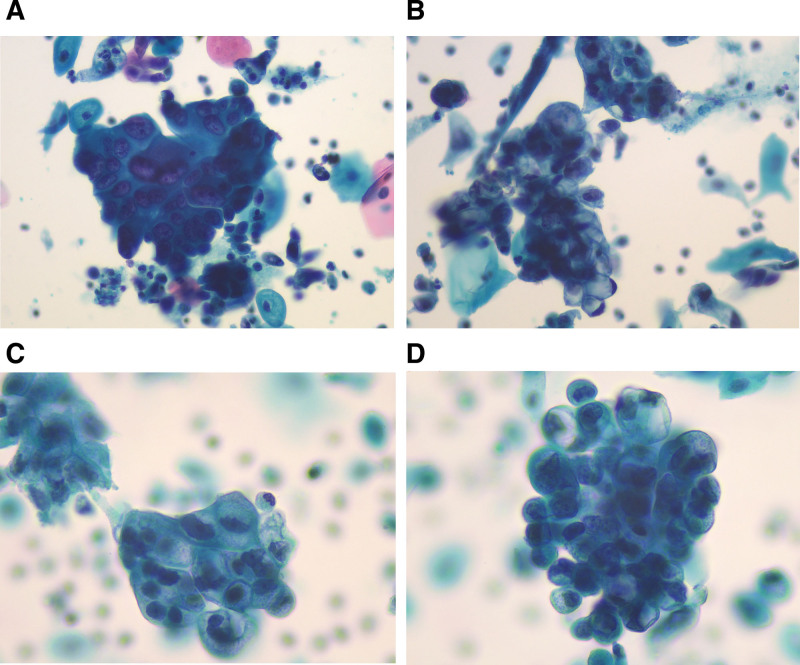
Cytologic manifestations of patients 2 and 3. (A and B) Cytologic manifestations of patient 2. The tumor cells were crowded into clusters, mucus was visible in the cytoplasm, and the cells were significantly heterogeneous with variable nuclear sizes. Distinct nucleoli were prominent (Pap staining, 400× magnification). (C and D). Cytologic manifestations of patient 3. The tumor cells were arranged in clusters, with mucus vacuoles in the cytoplasm, and the nuclei were significantly heterogeneous, with obvious nucleoli (Pap staining, 400× magnification).

The second patient was a 63-year-old female who presented with irregular postmenopausal vaginal bleeding. Uterine MRI revealed an abnormally-enhanced mass in the cervical region of the lower uterine segment, the largest cross-section of the lesion was about 5.8 cm × 3.9 cm. The lesion involved the endometrium and the upper third of the vagina, the cervical canal was obviously narrowed, and cervical cancer was considered. Fluids were identified in the uterine cavity along with small cysts in the cervix, the HPV test results were negative, and the LBC was diagnosed as AGC-FN. Microscopically, the tumor cells were crowded into clusters, mucus was visible in the cytoplasm, and the cells were significantly heterogeneous with variable nuclear sizes, obvious nucleoli, and dark nuclear chromatin (Fig. 2A and B). The pathological diagnosis based on the cervical biopsy was HPV-independent cervical adenocarcinoma of the gastric type. Microscopically, tumor cells were observed to grow infiltratively in the cervix, with some of the glands being well differentiated and pale-stained cytoplasm and nuclei located at the base. Some glands had enlarged nuclei with heterogeneous nuclei and distinct nucleoli. Immunohistochemistry showed MUC6 (+), P16 (−), ki-67 (10%, +), and the patient was administered a chemotherapeutic regimen but did not undergo follow-up.

### 2.4. Case 3

The third patient was a 59-year-old woman with irregular postmenopausal vaginal bleeding. MRI of the cervix revealed that the uterus was enlarged and the cervix was significantly thickened, with the largest lesion measuring about 4.4 cm × 3.5 cm × 3.8 cm. The lower margin involved the upper part of the vagina and did not reach the lower third of the vagina, nor did it involve the neighboring organs or the pelvis. Fluid was found in the uterine cavity along with multiple cystic foci in the cervix. The HPV test results were negative, and the LBC was diagnosed as AGC-FN. Microscopically, the tumor cells were arranged in clusters, mucus vacuoles were observed in the cytoplasm, and the nuclei were significantly heterogeneous (Fig. 2C and D). The pathological diagnosis based of the cervical biopsy was GAS, and microscopically, scattered infiltrative single cells or angulated glands were observed near glandular lesions. Immunohistochemically, the neoplastic cells were positive for MUC6, P16, and ki-67 (10%, +). The patient did not undergo any surgery, radiotherapy, or chemotherapy and was treated with traditional Chinese medicine and follow-up.

**Figure 3.**
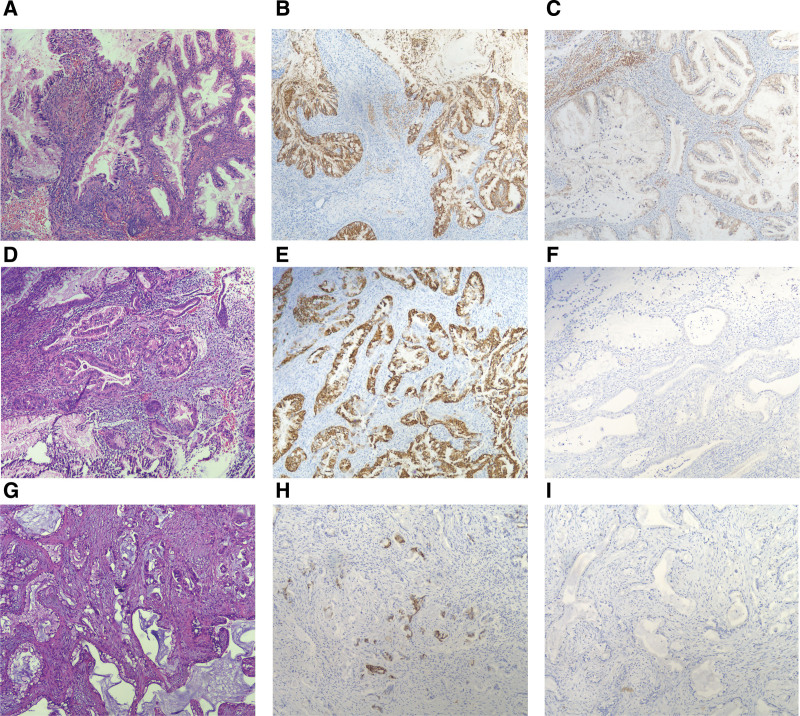
Histomorphology of gastric-type adenocarcinoma in 3 patients. Tumor cells in patients 1 (A), 2 (D), and 3 (G) exhibited irregular distribution and grew infiltratively in the cervix, with reactive hyperplasia of the surrounding fibrous interstitium (hematoxylin and eosin [H&E] staining, 100× magnification). The tumor cells in patient 1 were positive for MUC6 (B) and weakly positive for P16 (C). The tumor cells in patient 2 were positive for MUC6 (E) and negative for P16 (F). The tumor cells in patient 3 were positive for MUC6 (H) and negative for P16 (I) (EnVision, 100× magnification).

The histology of the 3 patients and the immunohistochemistry results for MUC6 and P16 are shown in Figure 3. Furthermore, clinical and cytological summaries of several articles and of our patients gastric adenocarcinoma of the uterine cervix are presented in Table 1.

**Table 1:** Clinical and cytological summary of gastric adenocarcinoma of uterine cervix.

First author [Reference number]	Clinical summary						Cytological summary			
	Number of cases	Year	Age (years)	Clinical presentation (n)	Stage (n)	Follow-up (n)	Cytology samples	Initial cytologic diagnosis (n)	Cytological characteristics	
									Golden-yellow mucin	Other features
Kudo R^[1]^	4	1990	35–51	Mucoid vaginal discharge (1), vaginal bleeding and lumbar pain (1), vaginal bleeding (2)	Ib (1), IIb (2), IIIb (1)	Dead (3), no evidence of disease (1)	Cytologic smears	suspected or diagnosed cytohistologically as having MDA	NA	Nuclei were irregular in size and shape honeycombs, palisades or sheets with glandular openings
Ishii K^[5]^	12	1999	41–70	Watery discharge (6); genital bleeding (1), watery discharge and genital bleeding (1), no symptoms (4)	NA	NA	Cervical/endometrial smear and imprint slides	NA	16 of 20 slides in cervical/endometrial smear were observed	Tumor cells derived from gastric metaplasia lack cellular atypia immunostaining with HIK1083 is a useful tool in the diagnosis
Hata S^[6]^	6	2002	45–64 (mean, 53.3)	NA	NA	Be well without recurrent disease (6)	CS	NA	All 6 cases were observed	Nuclei were more enlarged, the chromatin texture was coarsely granular
Kawakami F^[7]^	14	2015	35–68 (mean, 56.5).	NA	IB1 (1), IB2 (1), IIA (1), IIB (10), IIIA (1)	Alive with disease (2), dead (6), alive and well (6)	CS	AGC (3), ADC (11)	6 of 14 cases in LBP samples were observed	Monolayered and honeycomb sheets, vacuolar and/or foamy cytoplasm, intracytoplasmic neutrophil entrapment, vesicular nuclei with prominent nucleoli
Okuyama R^[8]^	2	2017	45 and 51	Watery vaginal discharge (2)	NA	NA	CS	NILM (2)	Two case were observed	Localization of mucin on the surface of glandular cell clusters was observed prior to nuclear atypia
Turashvili G^[9]^	17	2019	29–83 (median, 52)	NA	NA	NA	CS	22 cytology specimens from 17 cases. AGC-NOS (4), AGC, favor ADC (2), suspicious for ADC (1), ADC (15)	NO	Arranged singly and in cohesive clusters; pleomorphic nuclei with finely granular/vesicular chromatin, conspicuous nucleoli; pale, foamy, vacuolated cytoplasm, well-defined borders
Omori M^[10]^	1	2018	57	Amount of watery vaginal discharge	IIB	Dead	CS	NA	One case observed	Varicolored mucin or chicken-wire-mesh-type clusters
Toyoda S^[11]^	1	2019	64	Genital bleeding	IB2	Alive for 51 months after the initial operation on the uterus.	CS	ADC	NA	Irregular enlarged sheet, well‐defined cell borders, cytoplasmic mucin. Glandular architecture, large nuclei, irregular chromatin distribution, and macronucleoli
Lu S^[12]^	23	2019	28–73 (mean:, 51).	Vaginal mucoid discharge (6), vaginal bleeding (n = 5), abdominal pain (2), identified by physical examination (2), ultrasound evaluation (7), all patients showed cervical enlargement or mass	I–II (4), III–IV (11)	Follow-up information was available in 12 patients. alive (5), alive with disease (2), recurrence (1), dead (4).	11 of 23 patients have cytology testing. in LBP	11 of 23 patients have cytology testing. AGC-FN (1), AGC-NOS (3), HSIL (1) NILM (5)	NO	Tall columnar epithelial cells with pale, foamy or vacuolated cytoplasm were mostly common, followed by well-defined cytoplasmic borders
Greenland NY^[13]^	4	2021	36–61	Incidental on TVH for AUB and adenomyosis (1), post-coital bleeding (1), Abnormal pap with AGC (2)	IB2 (1); N/A. (1) IB3 (1); IB2 (1)	alive and well (3), alive with disease (1)	Cervical cytology samples were prepared using SurePath	Negative (2), AGC (2),	Only 1 case was observed	The study highlights the importance of noting a markedly increased number of bland-appearing endocervical cells on cervical cytology
Ryu A^[14]^	8	2021	24–76 (mean, 50.3)	NA	IB1 (3), IB2 (3), IB3 (1), IVB (1)	Alive (7), dead (1)	CS and LBC	AGC-NOS (2), AGC-FN (2), AIS (1), ADC (30)	3 of 8 on CS and 2 of 8 in LBC samples were observed	Amount of cell clusters, well-defined cell borders, fine chromatin pattern and pronounced nucleoli of GAS were more distinct on CS
Cho H^[4]^	14	2023	38–79 (mean, 56)	Tumors extended into the vagina and parametrium	I (2), II (4), IIIC1 (4), IIIC2 (2), IV (2)	Alive (9), died (5)	18 specimens from 14 cases on CS and in LBP	AGC-NOS (1), AGC-FN (1), malignant cells (9), AIS (2), ADC (5)	Only 1 case was observed	Flat, honeycomb-like cellular sheets, foamy or vacuolated cytoplasm, vesicular chromatin, and prominent nucleoli
Lee KH^[15]^	1	2024	74	General weakness and abnormal renal function tests	N/A	N/A	Voided urine and Cervical cytology in ThinPrep	Atypical cells, not otherwise specified	NO	Abundant cytoplasm, mildly pleomorphic nuclei, mild nuclear hyperchromasia, nuclear membrane
Our cases report	3	2024	57–63	Abnormal uterine bleeding and vaginal discharge (1), vaginal bleeding (2)	IIB (1) IIIA (2)	No recurrence (1), failure to follow-up (2)	LBC	4 slides from 3 cases, NILM (1), AGC-FN (3)	NO	“Drunken honeycomb” arrangement, especially three-dimensional spheres

ADC = adenocarcinoma, AGC-FN = atypical glandular cells-favor neoplastic, AGC-NOS = atypical glandular cells, not otherwise specified, AIS = adenocarcinoma in situ, CS = conventional smear, LBC = liquid-based cytology, LBP = liquid-based preparations, MDA = minimal-deviation adenocarcinoma, n = number of cases, NA = not available, NO = not observed.

The 3 patients underwent high-risk HPV testing using the Aptima HPV assay (Hologic, San Diego). LBC was used for cytological evaluation and all cytology samples were prepared using LBC (Anbiping, Guangzhou, China) and Papanicolaou (Pap) staining. Histology was assessed by hematoxylin and eosin staining, and all sections were immunostained with ready-to-use primary antibodies against MUC6, P16, P53, ki-67, CK7, CEA, CDX-2, P53, PAX-8, Ki-67, CK20, SATB2, ER, HNF-1β, and Napsin A (Zhongshanjinqiao, Beijing, China).

## 3. Discussion

Gastric-type cervical glandular lesions include benign, premalignant, and malignant lesions.^[16]^ Benign lesions include simple gastric metaplasia and lobular endocervical glandular hyperplasia (complex gastric metaplasia), postulated premalignant lesions comprise atypical lobular endocervical glandular hyperplasia and gastric-type adenocarcinoma in situ, and GAS is a malignant lesion.^[16–18]^

Omori M, Kondo T, Nakazawa K, et al classified gastric-type cervical glandular lesions into 3 categories, based on cytological morphology and cytoplasmic color. They recommended describing atypical endocervical cells with gastric-type mucin in cytology reports to improve the cytological screening for HPV-negative cervical cancer.^[19]^ Furthermore, several studies have reported that in conventional Pap stain cytology preparations, golden-yellow mucins are one of the most important features of gastric-type glandular lesions.^[5,6,8,10]^

No significant golden-yellow mucins were observed during LBC preparation. In a retrospective study, only 3 of 15 LBC preparations in patients with GAS had gold-colored cytoplasm; thus, yellow mucin is an uncommon Pap test result, and a presumptive diagnosis of GAS can only be made in the context of appropriate clinicopathology.^[20]^ Omori M, Kondo T, Nakazawa K et al also noted that “it is difficult to identify yellow mucin on LBC because the mucin becomes lighter in color.^[19]^” Some researchers have observed that golden-yellow mucin was not obvious in the cytoplasm of tumor cells, which suggests that golden-yellow mucin may not be a particularly sensitive feature in LBC.^[4,12–14]^ Other scholars did not appreciate this golden-yellow mucin in their samples, which may relate to differences in staining reagents and protocols.^[9,15]^ Therefore, the value of atypical endocervical cells with gastric-type mucin reporting terminology and its categorization in the application of different staining patterns deserve further discussion.

The recognition of GAS should focus on observing the morphological features of the cells. The cytological features of GAS include monolayer and honeycomb-like lamellae, foamy cytoplasm, intracytoplasmic neutrophils, and vesicular nuclei with different nucleoli.^[7]^ High columnar cells, cytoplasm moderately to abundant mucin, well-defined cytoplasmic borders, finely granular chromatin, and a low nucleoplasmic ratio are also diagnostic features.^[11,12]^

In the first LBC sample from patient 1, some of the glandular epithelial cells were flat, while some were clustered and globular. The polarity within the glandular cell clusters was disorganized and arranged in a “drunken honeycomb” pattern, which is a cytological feature of well-differentiated mucinous adenocarcinomas. Mucinous tumor cell clusters show well-defined cell borders and unevenly distributed nuclei with unequal spacing and consist of monotonous high columnar epithelial cells, abundant cytoplasmic mucin, and nuclei located in the basal part of the cells.^[3]^

We observed that some of the cells were three-dimensional spherical, which is a special form of the “drunken honeycomb” arrangement. The cells were also characterized by abundant cytoplasm, distinct cell boundaries, uneven distribution of nuclei, unequal spacing of cells, and nuclei located at the base. This three-dimensional spherical structure was suggestive of tumors with mild cellular heterogeneity. Patients 2 and 3 had a high clinical stage and moderately-to-poorly differentiated cervical tumor cells, with significant cellular heterogeneity and structural abnormalities.

Owing to the complexity and variety of GAS morphological features, it should be distinguished from normal glandular epithelium and usual-type endocervical adenocarcinoma, mainly by cytology. Normal cervical columnar epithelial cell clusters are spread in a monolayer, with honeycomb and brush border. The nuclei are roughly equally spaced and not enlarged or heteromorphic, with normal chromatin. The usual-type endocervical adenocarcinoma is formed by dense clusters of deeply stained cells, with an overlying crowded arrangement of columnar cells with enlarged nuclei, coarse granular chromatin, dark staining, mitotic images, apoptotic vesicles, visible large nucleoli, and a tumor background.

GAS is prone to metastasis, has a poor prognosis, and is relatively resistant to chemotherapy; therefore, it is important to accurately identify GAS based on its cellular morphology in the early stages of the disease.^[21,22]^ From our findings in this series of cases, we believe that the “drunken honeycomb” cellular arrangement is of diagnostic importance, especially when three-dimensional spheres are present, and may be a characteristic cytological finding of well-differentiated GAS.

## Acknowledgments

We would like to thank Editage (www.editage.cn) for English language editing.

## Author contributions

**Conceptualization:** Chang Zeng.

**Methodology:** Anchun Liu, Maiqing Yang, Hao Zou, Xiaolin Gong, Chang Zeng.

**Writing – original draft:** Anchun Liu, Maiqing Yang, Hao Zou, Xiaolin Gong, Chang Zeng.

**Writing – review & editing:** Anchun Liu, Maiqing Yang, Hao Zou, Xiaolin Gong, Chang Zeng.
